# Applying precision medicine principles to the management of multimorbidity: the utility of comorbidity networks, graph machine learning, and knowledge graphs

**DOI:** 10.3389/fmed.2023.1302844

**Published:** 2024-01-24

**Authors:** Richard John Woodman, Bogda Koczwara, Arduino Aleksander Mangoni

**Affiliations:** ^1^Flinders Health and Medical Research Institute, College of Medicine and Public Health, Flinders University, Adelaide, SA, Australia; ^2^Department of Medical Oncology, Flinders Medical Centre, Southern Adelaide Local Health Network, Adelaide, SA, Australia; ^3^Department of Clinical Pharmacology, Flinders Medical Centre, Southern Adelaide Local Health Network, Adelaide, SA, Australia

**Keywords:** network analysis, graph database, disease comorbidity networks, multimorbidity, knowledge graphs, graph machine learning, precision medicine

## Abstract

The current management of patients with multimorbidity is suboptimal, with either a single-disease approach to care or treatment guideline adaptations that result in poor adherence due to their complexity. Although this has resulted in calls for more holistic and personalized approaches to prescribing, progress toward these goals has remained slow. With the rapid advancement of machine learning (ML) methods, promising approaches now also exist to accelerate the advance of precision medicine in multimorbidity. These include analyzing disease comorbidity networks, using knowledge graphs that integrate knowledge from different medical domains, and applying network analysis and graph ML. Multimorbidity disease networks have been used to improve disease diagnosis, treatment recommendations, and patient prognosis. Knowledge graphs that combine different medical entities connected by multiple relationship types integrate data from different sources, allowing for complex interactions and creating a continuous flow of information. Network analysis and graph ML can then extract the topology and structure of networks and reveal hidden properties, including disease phenotypes, network hubs, and pathways; predict drugs for repurposing; and determine safe and more holistic treatments. In this article, we describe the basic concepts of creating bipartite and unipartite disease and patient networks and review the use of knowledge graphs, graph algorithms, graph embedding methods, and graph ML within the context of multimorbidity. Specifically, we provide an overview of the application of graph theory for studying multimorbidity, the methods employed to extract knowledge from graphs, and examples of the application of disease networks for determining the structure and pathways of multimorbidity, identifying disease phenotypes, predicting health outcomes, and selecting safe and effective treatments. In today’s modern data-hungry, ML-focused world, such network-based techniques are likely to be at the forefront of developing robust clinical decision support tools for safer and more holistic approaches to treating older patients with multimorbidity.

## 1 Introduction

Multimorbidity, defined as the coexistence of two or more diseases in one individual, is a major health challenge globally because of its high prevalence (1, 2), complex care needs (3) and association with inferior healthcare outcomes (4–6). Current professional guidelines for disease management for patients with multimorbidity typically follow a single-disease approach to care that is either not adapted to the needs of persons with multimorbidity (7) or is poorly adhered to due to treatment guideline complexity (8). As a consequence, patients with multimorbidity often take five or more different medicines simultaneously, an accepted definition of polypharmacy (9, 10), and have a greatly increased risk of medication interactions, adverse drug reactions (ADRs), and poor health outcomes and quality of life (7, 11–13). Compounding the problems of using a single-disease framework for care in this population is the fact that most guidelines are also based on empirical evidence obtained from randomized controlled trials (RCTs) using strict inclusion and exclusion criteria that exclude patients with multimorbidity, thereby perpetuating the lack of evidence for treating this highly complex and heterogenous population (14–16).

Personalized, or precision medicine, is a tailored approach to patient care where patients are stratified based on their clinical profile, with the key idea being that medical decision-making is based on individual profiles that also include clinical, molecular, and behavioral biomarkers (17). This approach has revolutionized the management of many conditions, most notably in oncology, but its use in the management of multimorbidity is limited (18), despite calls for more holistic and personalized approaches to prescribing (11). A limitation in its development has been a lack of availability or identification of appropriate methodologies that can fully harness the available information from highly interconnected multimodal sets of data. However, promising approaches to achieving precision medicine for multimorbidity have recently been identified and further developed. The application of graph databases to disease networks is gaining increased popularity as a method of studying complex disease relationships due to their natural ability to allow an intuitive visualization of heavily interconnected data and their increased performance and flexibility compared to using more traditional relational databases (19). Heterogeneous graph networks can be used to integrate knowledge from different medical domains, including diseases and drugs, and incorporate their complex interactions (20), and machine learning (ML) methods, including graph neural networks, are now being applied to multimorbidity disease networks to improve disease diagnosis, treatment recommendation, and patient prognosis (21, 22).

In a landmark study in multimorbidity network analysis, a phenotypic disease network was created from the ICD-9 codes of more than 32 million inpatient claims to study disease progression (23). A wide range of disease connectivity existed, with illnesses progressing along the disease network and progression differing by gender and race. Such disease progression can be expected given that disease networks reflect underlying disease pathways and pathologies, with many diseases sharing common genes, proteins, environmental factors, and biological pathways (21, 24–27). The fact that patients develop diseases in phenotypic networks that are close to those they already have rather than by chance alone (28) also supports the concept of underlying molecular mechanisms that facilitate (or prevent) disease occurrence (28). Importantly for precision medicine, since proteins and genes associated with a specific disease tend to also cluster in the same network neighborhood, diseases driven by perturbations of these components are therefore also phenotypically similar, leading to similar responses when targeted by a therapeutic (20). Potential disruption of the disease network can also be achieved by targeting the network’s “hub” diseases for specific intervention (25, 28, 29) since such “hubs” are associated with patient outcomes (30) and the proteins that represent the disease “hubs” likely have a special biological role (29).

**Figure 1:**
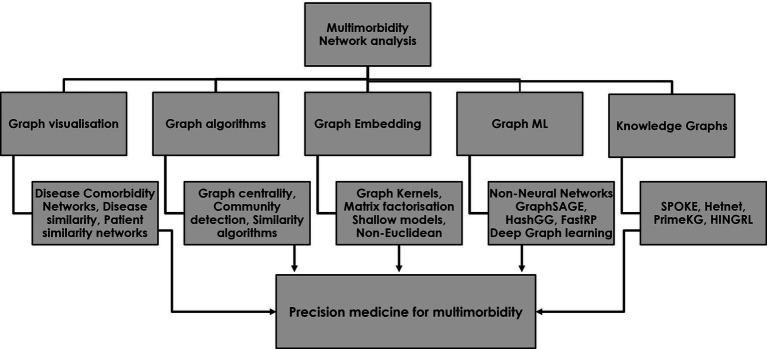
The use of network analysis for studying multimorbidity and developing a precision medicine approach to safe and effective prescribing for multimorbidity. The five major categories of network analysis each contribute to a precision medicine approach in multimorbidity.

Establishing disease phenotypes and disease hubs within disease comorbidity networks are specific examples of many different approaches that exist for extracting information from multimorbidity networks in ways that can improve our understanding of complex disease–drug–patient networks and support holistic and personalized prescribing in multimorbidity (31). As a first step in the process, the visualization of disease–drug–patient information in the form of graphs provides an immediate and intuitive interpretation of different medical entities within a complex disease network whilst providing important context to the relationships. Beyond visualization, network analysis provides a range of powerful tools for understanding the complex structure of multimorbidity and for improving disease diagnosis and treatment. These include the extraction of graph features, graph embedding methods, graph ML, including graph neural networks for making predictions on unseen data, and knowledge graphs to uncover hidden relationships (Figure 1).

Table 1 provides an overview of the use of different methods commonly applied in the setting of disease comorbidity network analysis, as well as an evaluation of their strengths and limitations. Feature extraction algorithms capture properties of the graph nodes, such as their importance, closeness, and community membership, generating novel graph features for improving prediction with downstream outcome models. Similarity algorithms, such as random walks and kernels, enable a better understanding of the structure of the network. Graph embedding techniques capture the latent topology of a graph, including node similarity in the form of vectors for use in prediction models (20), and graph ML enables fully end-to-end models with graph data as input and node, edge, or subgraph prediction as output for data outside of the existing network (44, 45). The use of graph neural networks (GNNs) to date includes predicting drug–drug interactions (46), modeling polypharmacy side effects, and learning the temporal patterns of disease development in comorbidity networks (47). Finally, knowledge graphs can be used to make use of the known connections between drugs, proteins, and genes to uncover new associations between different entities for use in areas such as drug repurposing and disease diagnosis.

**Table 1:** Methods used in network analysis and their uses, strengths, and limitations for disease comorbidity networks.

Method	Uses	Strengths/Limitations	Example study
Network creation Graphing packages and libraries Gephi, Neo4j, Python libraries: NetworkX, igraph	Bipartite disease network creation	Describes overall entities and complexity of their relationships	Predicting high-cost patients using a DCN with Gephi software and igraph for community detection (32).
Similarity algorithms Jaccard similarity score, Overlap coefficient	Unipartite projection Measuring graph similarity.	Describes the indirect connections between diseases. Also measures their strength.	Bipartite graphs in systems biology describing the projection process in detail (33)
Community detection algorithms Louvain Label propagation Walktrap Girvan-Newman	Phenotyping, subgraph detection	Automatically detect graph modules containing sets of nodes that cluster locally. Different algorithms may detect different clusters.	Structural knowledge analysis and modeling of multimorbidity using graph theory-based techniques (34)
Feature extraction algorithms Centrality algorithms: Degree centrality Eigenvector centrality Page rank Clustering coefficient	Describes network structure and identifies key nodes. Generation of novel graph features useful for disease prediction.	Requires building separate graphs for separate disease populations. Useful for disease prediction using supervised ML algorithms in the same population.	A PSN created from unipartite projection extracted several different centrality metrics that were all predictive of type 2 diabetes (35).
Graph embedding algorithms Kernels: k-walk, shortest path, Weisfeiler–Lehman (WF). WF isomorphism test for assessing differences between graphs (36). Non-negative matrix factorization (NNMF) (37)	Subgraph detection (graph kernels) and dimensionality reduction (NNMF) for node embedding. Network comparison (WF test).	Early methods used for node embedding. Difficult to learn node embeddings with large graphs.	Test for differences in network structure of physiological variables during COVID-19. The clustering coefficient was disrupted (38). Aging and diseases changed the topology of the networks.
Shallow embedding methods Diagnosis to Vector (Dx2vec) Metapath2vec (39) PageRank (Google)	Modern node embedding and classification algorithms.	Non-inductive: cannot build a model for application to new data points. Ignores information of node properties	Predicting self-harm incorporating temporal diagnosis sequences (40).
Inductive graph ML models HashGNN GraphSAGE (41)	Node embedding, plus node and link prediction on unseen data.	The graphs used for prediction must be reasonably similar to those used for training.	Predicting cellular functions from protein–protein interaction graphs (41).
Graph neural networks Graph convolutional networks (GCN) Graph attention networks (GAN) Jumping knowledge network (JK-Net) Message passing neural networks (MPNN) Decagon algorithm; GCN for multi-relational link prediction.	Inductive graph ML techniques.	Captures the higher order relationships within a graph. High complexity May not scale well. Low interpretability and explainability.	Modeling polypharmacy side effects (42).
Knowledge graphs	Knowledge discovery	Open-source datasets created from publicly available datasets.	Hetionet: 47,000 nodes and 136 diseases Drug repurposing (43). Disease prediction Treatment recommendation

ML, machine learning; DCN, disease comorbidity network; PSN, patient similarity network.

The aim of this article is to provide a comprehensive overview of promising methodological approaches that could be applied to the management of multimorbidity. Specifically, we (1) describe the basic concepts of creating patient–disease, disease–disease, and patient–patient networks and (2) describe the main features and use of graph algorithms, graph embedding methods, graph ML, and knowledge graphs for the study of patients with multimorbidity. Together, these enable network visualization, characterization of network structure, node embedding for downstream prediction, and transductive and inductive graph ML algorithms for end-to-end prediction using graphs as input data. Their ability to incorporate information from different medical domains, determine graph structure, and assist in disease phenotyping, prediction, and treatment recommendation open the gateway to the development of tools that can realistically provide robust clinical decision support tools for safer and more holistic approaches in the treatment of patients with multimorbidity.

## 2 Literature search

A non-exhaustive database search strategy was developed that identified relevant literature examples of network analysis being used in the study of multimorbidity. We searched the databases of PubMed Central, Semantic Scholar, Google Scholar, and arXiv (Cornell University) from inception to 31 August 2023 using the terms graph, network, graph database, network analysis, graph machine learning, graph representation learning, and knowledge graphs combined with the terms multimorbidity and comorbidity for selecting the study population of interest. We included network studies that were focused on either comorbidity, multimorbidity, or treatment for multimorbid populations, especially those developing unipartite disease comorbidity or patient similarity networks to develop improved prediction via the use of graph features or graph ML. We excluded studies that were not of an applied nature, review articles, and studies that were not focused on either improving prediction or precision medicine in a multimorbid population. For knowledge graphs, we selected publications linking diseases to drugs for the purpose of drug repurposing or precision medicine. The initial database search strategy is described in more detail in Supplementary Figure S1.

## 3 Network creation

### 3.1 Bipartite patient-disease networks

**Figure 2:**
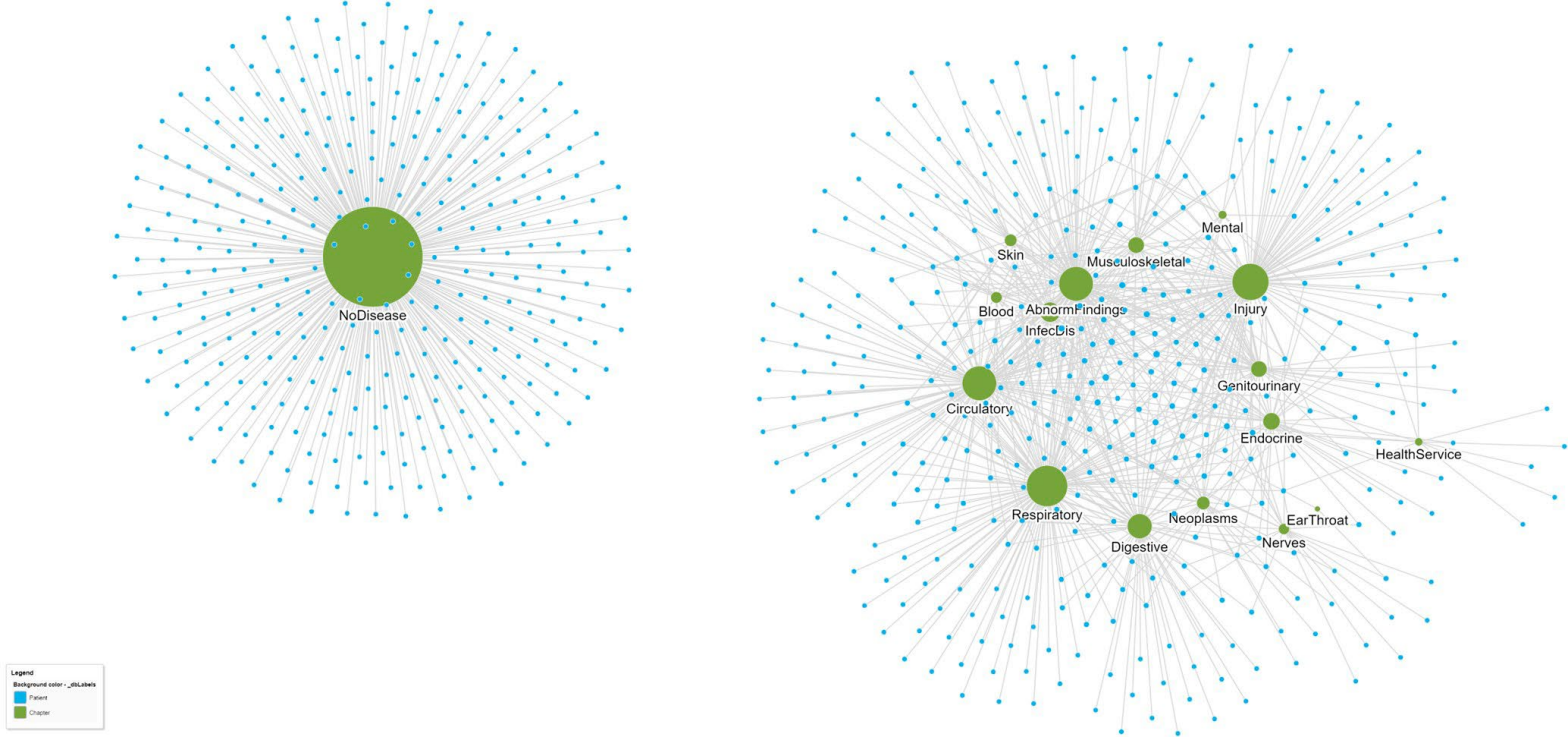
A bipartite graph network of patients (small blue circles) and their ICD-10 disease chapters (larger green circles). Patients who are closer to one another are more similar. For example, patients at the center of the network share more diseases than those at the edge of the network. Similarly, ICD-10 disease chapters that are closer together are also more similar since they tend to co-occur in patients more often. From this single bipartite network, separate disease–disease and patient–patient networks can be created that reflect disease and patient similarity, respectively. The data are from a set of *n* = 737 patients attending a hospital geriatric ward (39).

Biological networks typically include more than one type of entity (proteins, genes, diseases) with edges defined by relevant types of relationships; for example, patients and their diseases might have a relationship type “has-disease” to link patient and disease nodes. An example of a disease–patient bipartite graph is shown in Figure 2, with patients connected to disease chapters defined by the International Classification of Diseases, 10th Edition (ICD-10). The existence of an edge that connects two nodes demonstrates that a patient has a disease within the ICD-10 chapter, and equally, the lack of an edge between nodes demonstrates the lack of any patient–disease relationship. Thus, the edges in the graph between the disease nodes (green) and patient nodes (blue) represent the disease diagnoses of the individual patients. A close inspection of the network reveals that patients in the center of the network have more comorbidities than patients at the edge of the network, and their closeness reflects a similarity in terms of both the number and nature of the diseases that they share. Among the disease chapter nodes, closer positioning of any two diseases reflects the increased likelihood of them being found in combination in the same patient than diseases that are more isolated from one another.

## 4 Similarity algorithms

### 4.1 Unipartite disease comorbidity network (DCN)

The proximity of diseases in a DCN likely reflects common disease-associated genes and shared molecular mechanisms and etiologies (27) including, for example, inflammation (48). Information on disease similarity within a disease network can therefore be used to assist in predicting disease and for developing precision medicine approaches to prescribing. To determine the presence and strength of disease–disease connections in the disease network, it is necessary to project the bipartite patient–disease network into a unipartite network consisting of only disease nodes and edge connections based on some way of measuring disease similarity. The first requirement for the presence or absence of an edge between two diseases is determined by whether any patient in the network has both diseases (49). The simplest and most widespread approach for extracting this edge backbone of bipartite projections is through the application of node similarity algorithms that compare all possible node pairs based on the nodes they are each connected to (33). An unconditional (or global) threshold weight is selected and applied to all edges in the unipartite projection, and edges are retained in the backbone network only if their weight in the unipartite projection exceeds this predefined threshold, which is most often set to zero. The node similarity algorithm can be applied using either the Jaccard similarity score, the cosine similarity score, or the overlap coefficient as the similarity metric (Box 1).

**Figure 3:**
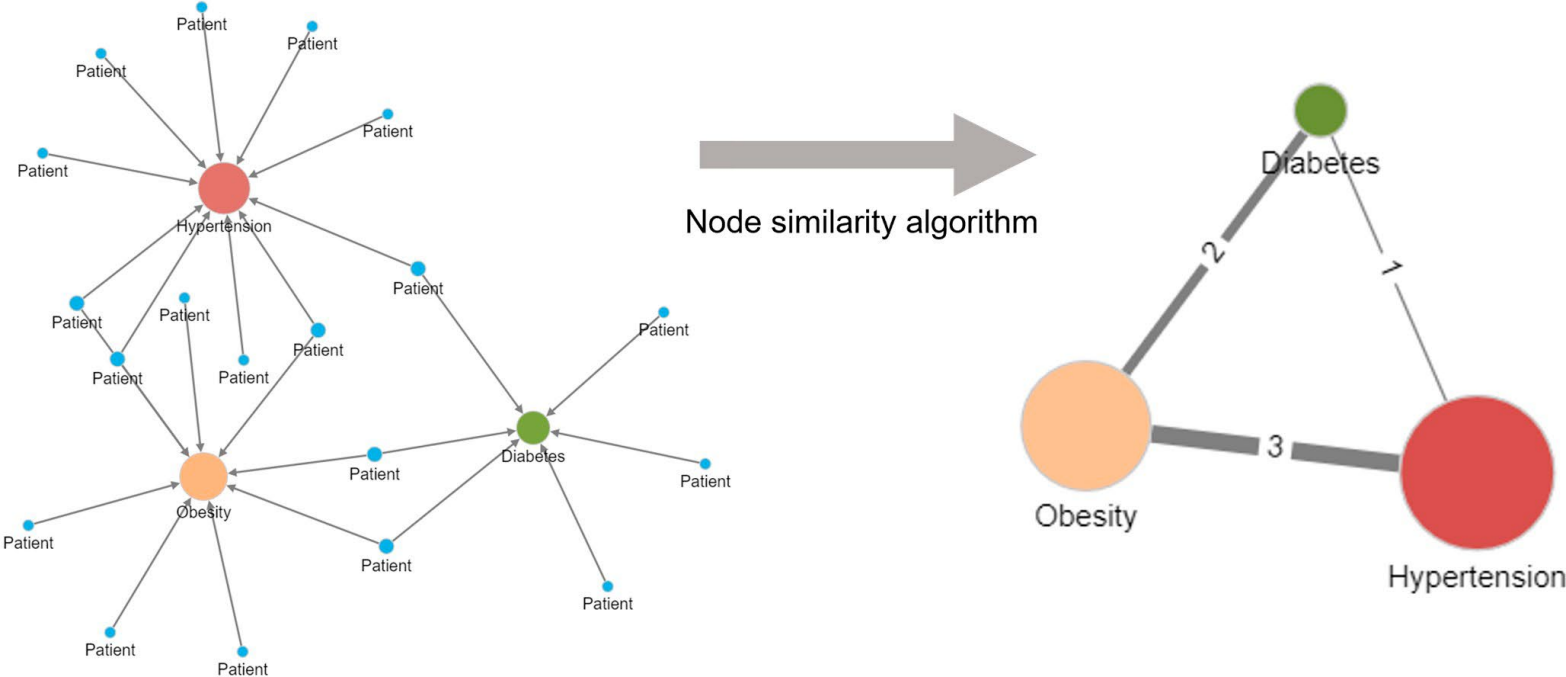
Creation of a unipartite disease–disease network from a bipartite disease–patient network using a node similarity algorithm. Diseases are indirectly related to one another in the bipartite patient network due to patients having more than one disease. More frequently shared disease pairs are more similar and are given a higher edge weight in the unipartite disease–disease network to reflect their stronger similarity. The node size reflects the number of patients with the named disease.

Figure 3 illustrates the application of the Jaccard similarity algorithm to five patients sharing four different diseases. The diseases are indirectly connected to one another due to patients having more than one disease. For example, one patient has both hypertension and diabetes. Within the network, the relationship strengths (edge weights) are based on the number of patients sharing the same pair of diseases.

**Figure 4:**
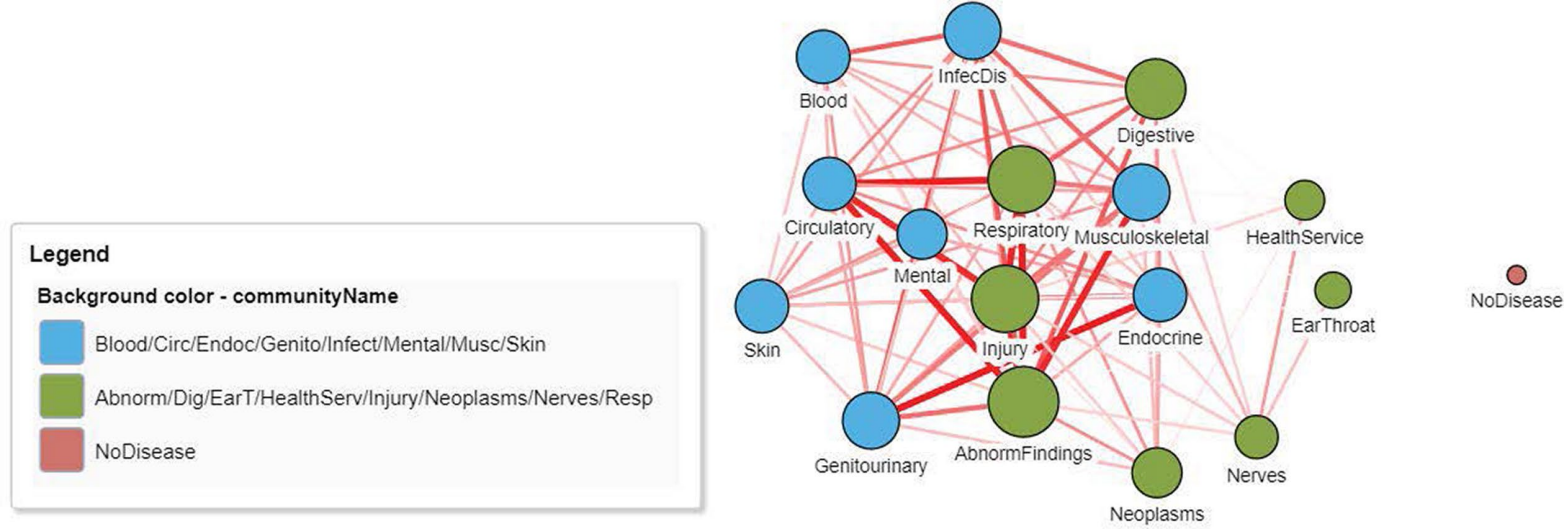
A DCN after application of a node similarity algorithm to the disease–patient network in Figure 2. The unipartite projection is created using the resulting Jaccard similarity scores, with all edges of the disease–disease network being retained (Jaccard similarity score > 0). The width and the color intensity of the network edges reflect the Jaccard similarity score. The color of the nodes is based on a community detection algorithm that identified two broad disease clusters among this geriatric population. The size of the nodes reflects the number of connections (degree centrality) for the disease chapter.

Figure 4 displays the DCN created from the real-world bipartite data in Figure 2 after the application of a bipartite projection using the Jaccard similarity score. All edges of the network have been retained by using a default Jaccard similarity cut-off score of ≥0. Some node pairs are more strongly connected, such as in the Circulatory and Abnormal Findings Disease chapters and the Genitourinary and Endocrine chapters. This is due to the fact that many patients share these disease pairs. Nodes toward the center of the network are typically more strongly connected and have a higher degree centrality since they are linked to more disease chapters. Some disease chapters, such as Health Services and Ear and Throat, are less well connected to other diseases and are therefore at the periphery of the network and have a smaller degree centrality.

BOX 1 **Node similarity metrics.**

The Jaccard similarity score and the Overlap coefficient measure node similarity based on their shared connections.

Jaccard similarity score

Given two vectors A and B used to represent node connections, the Jaccard Similarity is computed using the following formula:

$$J\left(A,B\right)=\frac{A\cap B}{A\cup B}=\frac{A\cap B}{\left|A\right|+\left|B\right|-\left|A\cap B\right|}$$

The overlap coefficient

The overlap coefficient is computed using the following formula:

$$O\left(A,B\right)=\frac{\left|A\cap B\right|}{\min\left(\left|A\right|,\left|B\right|\right)}\;$$

The cosine similarity

The cosine similarity score is computed using the following formula:

$$S_{c}\;\left(A,B\right)=\frac{A\cdot B}{A\cdot B}=\frac{\sum_{i=1}^{n}A_{i}B_{i}}{\sqrt{\sum_{i=1}^{n}{A_{i}}^{2}}\cdot \sqrt{\sum_{i=1}^{n}{B_{i}}^{2}}}$$

### 4.2 Node similarity metrics

After establishing a network that contains all possible edges, further selection of which edges to retain is typically performed to eliminate disease–disease connections that are relatively weak and to focus instead on the most important disease connections to improve visualization and understanding. The use of the similarity cut-off score ≥ 0 is one approach and includes setting the cut-off at a percentage of the maximum or at the mean similarity score. However, this is a somewhat arbitrary approach, and therefore more formal measures have been developed based on statistical metrics and significance. Box 2 describes some commonly used metrics used for measuring edge strength and determining the selection process. Each approach includes some form of adjustment to account for the prevalence of each disease (49). Two commonly used measures of edge strength are the relative risk (RR) and the Phi (ϕ) correlation. These each have their separate advantages and disadvantages, including biases toward either rare or highly prevalent diseases (32), and are therefore sometimes presented together. For example, when creating the phenotypic disease network (PDN) to explore disease progression using the ICD-9 codes from more than 32 million inpatient claims, the strength of comorbidity relationships was quantified using both the RR and ϕ correlation, with edges retained based on a RR > 20 or a ϕ correlation >0.06 (23). The same approach was used when developing a comorbidity network to predict the risk of diabetes among hospital patients with the strength of the co-occurrence among diseases, resulting in 618 disease connections using a RR > 20 and 2,515 disease connections for ϕ > 0.06 among 330 ICD-9 disease codes (50). The Phi correlation was trialed with different levels of statistical significance when developing comorbidity networks in the EpiChron study of patients with chronic obstructive pulmonary disease (COPD) and congestive heart failure (CHF) (28).

The disease co-occurrence correlation (CC) also attempts to reduce the potential bias created by disease prevalence and was used when generating a disease co-occurrence network to predict high-cost patient encounters on hospital admission (32). There remained 38,812 statistically significant pair-wise co-occurrence relationships among 2025 diagnoses at a network density of 0.019. The propensity score for being a high-cost patient was based on the CC_xy_ edge weights and was predictive of high-cost patients (32).

However, a limitation of all the above approaches for determining edge strength is the lack of conditioning for other diseases beyond the pair being considered. To overcome this, the log odds ratios for disease–disease pairs can be calculated using separate logistic regression models with the elastic net regularization penalty to limit the strength of the coefficients. Each model creates a single row of P-1 coefficients for a P × P disease–disease edge matrix. When compared to non-conditioned edge weights, the conditioned network was smaller (509 vs. 589 disease nodes), easier to interpret, and associations appeared more clinically insightful. The edge density of the network was 0.02, the global transitivity was 0.24, the diameter was 10, and the average distance was 3.63 (51).

BOX 2 **Statistical approaches for measuring edge strength.**

Disease-disease relative risk

For a comorbidity network, the RR of observing a pair of diseases i and j affecting the same patient is given by:

$$RR_{ij}=\frac{C_{ij}N}{P_{i}P_{j}}$$

Where, $C_{ij}$ is the number of patients affected by both diseases, *N* is the total number of patients in the population and $P_{i}\;$ and $P_{j}$ are the prevalence of diseases i and j.

Disease-disease ϕ-correlation

For a disease comorbidity network, the Phi-correlation, which is Pearson’s correlation for binary variables, can be expressed mathematically as:

$$\phi_{ij}=\frac{C_{ij}N-P_{i}P_{j}}{\sqrt{P_{i}P_{j}\;\left(N-P_{i}\right)\left(N-P_{j}\right)}}$$

Disease co-occurrence correlation

The formula for the co-occurrence correlation (CC) of two diseases, x and y is:

$$CC_{xy}=\frac{\sqrt{2}C_{xy}}{\sqrt{P_{x}^{2}+P_{y}^{2}}}$$

Where, $C_{xy}$ is the co-occurrence of disease x and y across patient encounters, Px and Py are prevalence of diseases x and y respectively.

Log odds ratio

For P diagnoses categories, a P×P weight matrix is created, with each off-diagonal element (logOR_ij_) representing the associations between diagnosis category i and diagnosis category j in the form of a log odds ratio. Using logistic regression with elastic net penalty (51), the logOR for some i, j pairs is set to zero indicating zero or undetectable associations.

### 4.3 K-nearest neighbor (k-NN) and handcrafted similarity features

Several other approaches also exist to capture the similarity of nodes based on the nodes in the neighborhoods and their edges. Algorithm metrics include K-nearest neighbor scores (52) which allow for the identification of similar patients based on patient properties such as clinical characteristics, laboratory data, medications, and disease diagnoses. Other patient similarity approaches have been used for predicting disease based on matching an individual’s disease network with that of a DCN, including for diabetes prediction (49) and for future diseases (50). For diabetes prediction, the graph node match score and the graph pattern match score, which assessed the similarity of nodes and edges, respectively, for a new patient with an existing diabetes network, were stronger predictors of diabetes than age, sex, and smoking/alcohol and provided an overall diabetes prediction accuracy of 86.22% (49). For future disease prediction, high levels of accuracy and recall (0.8593 and 0.4903, respectively) were obtained using measures of support and confidence from associative rules analysis (50).

## 5 Community detection

Following the creation of the DCN, community detection algorithms can be applied to better understand the structural properties of the network and to elicit heterogeneous patient groups, an essential component of precision medicine. The existence of underlying shared pathophysiology results in many biological networks showing a high degree of natural clustering, with highly interlinked local regions in the network known as either modules, groups, or communities (33). Detecting and characterizing these modules is one of the most widely used applications in network analysis, and in biological networks, it can help explain the development and complex nature of biological systems (53, 54). Detecting these modules helps identify disease phenotypes, highlight opportunities for intervention and/or screening, and study the multimorbidity patterns that underlie primary diagnoses such as depression (48, 55), CHF (28), and COPD (56).

### 5.1 Modularity and community detection algorithms

Modularity is a common network metric used to describe the extent of clustering within the network and is defined as the fraction of the edges that fall within the given groups of nodes minus the expected such fraction if edges were distributed at random. Values range from −1 to +1. If positive, then the number of edges within groups exceeds the number expected based on chance (30) indicating the presence of community structure. A range of community detection algorithms exist with different approaches used to identify clustering, including using edge-betweenness (Girvan–Newman), neighboring node labels (label propagation), maximizing the local modularity score (Louvain), and random walks (Walktrap). Since optimizing the modularity is a highly effective approach for detecting the possible divisions of a network (57), this was the basis for creating the Louvain algorithm (58). In a comparison of the Louvain, label propagation, Walktrap, and Girvan–Newman algorithms for clustering a large disease comorbidity network (34), the label propagation algorithm detected more than two times the number of communities than the Louvain, highlighting the value of considering multiple algorithms to fully examine network structure. In addition, aging also increased the number of clusters, revealing an increase in the different types and layers of multimorbidity burden that occur with aging (34). DCNs can have very high levels of modularity, reflecting the high degree of disease clustering among patients and the presence of disease phenotypes. Using a dataset of hospital admissions from Madrid, Spain, modularities ranging from 0.78 to 0.90 were observed, containing up to 60 different disease diagnosis communities (34). When predicting high-cost patients, after retaining edges that were significant, 120 non-overlapping communities were detected among 653 disease nodes using the Louvain algorithm, which included nine major disease groups (32).

To further assist with phenotyping, clinical measures are sometimes added as a separate node type into the disease–disease network prior to applying community detection algorithms. This approach was used to determine COPD phenotypes using 10 clinically relevant variables (including age and forced expiratory volume) added to a network of 79 comorbidities (30). A community detection algorithm identified four modules that reflected meaningful syndromic patterns of COPD (older cardiovascular, younger current smokers with behavioral risk factors and psychiatric conditions, mild–moderate airflow obstruction with metabolic syndrome including high body mass index [BMI], gastro-esophageal reflux, osteoporosis, and degenerative joint disease), suggesting an opportunity for targeted screening (30). Additionally, the four modules identified among the non-COPD controls had distinctly different clinical phenotypes (cardiovascular, anxiety, and depression; older with cardiovascular risk; and high BMI with obstructive sleep–apnea).

### 5.2 Other clustering methods

#### 5.2.1 Local clustering coefficient

Separately from community detection, which measures the overall level of clustering in a network, the level of local clustering around each node can be measured using the local clustering coefficient. This quantifies how likely it is that the neighbors of a node are also connected. The clustering coefficient of a node u is:


$$C_{u}=\frac{2\;T\;\left(u\right)}{\deg\left(u\right)\;\left(\deg\left(u\right)-1\right)}$$

where *T*(*u*) is the number of triangles through node *u* and deg (*u*) is the degree of *u*. It is based on the triangle count, where a triangle is a set of three nodes in which each node is related to the other two nodes. Triangle counts can also be used to detect communities and measure their cohesiveness. In a univariate patient–patient network with diabetes and non-diabetes patients, there were 38 tightly connected communities, and the clustering coefficient was a significant predictor of future diabetes (35).

#### 5.2.2 K-nearest neighbors

The K-nearest neighbors (K-NN) algorithm compares the given properties of each node, and the k nodes where these properties are most similar are the k-nearest neighbors. The input of this algorithm is a homogeneous graph and does not need to be connected. Instead, relationships are created between each node and its k-nearest neighbors, and a distance value for all node pairs in the graph is calculated based on node properties, for example, a patient’s age. When predicting length of stay (LOS), aggregated LOS functions (mean, SD, min, and max) were calculated for each patient using their K = 100 nearest neighbors in a patient similarity network and then used for predicting LOS (59).

#### 5.2.3 Hierarchical clustering

The identification of clusters using hierarchical clustering based on similarity scores is an alternative way to reduce the dimensionality of datasets for predicting health outcomes, ensure adequate separation of clusters, and enable varying the number of clusters. This approach was used for predicting diabetes readmission and the severity of CHF (60). First, a DCN was created using the Jaccard similarity score for ICD-9CM codes, and then a distance matrix for the disease codes was created using the formula Distance D_A,B_ = 1 − S_A,B_ where S_A,B_ is the similarity score for nodes A and B. The distance matrix was used as the dataset for hierarchical clustering using the inverse variance method. Patients were then given binary codes for each disease that matched a disease cluster. Using between 5 and 40 clusters considerably increased predictive accuracy for heart rate and blood pressure outcomes in CHF patients, with gains of between 10.7 and 22.1% in predictive accuracy for CHF severity of condition prediction and 4.65–5.75% in diabetes readmission prediction.

#### 5.2.4 Temporal phenotyping

To predict incident CHF and 1-year hospitalization among patients with CHF and COPD, novel temporal graph phenotypes were created by combining disease diagnosis and drug class data (61). Nodes in the graph represented medical events in the electronic health record (EHR), including disease diagnosis and drug prescribing, and directed edges represented the temporal sequence between events, which were weighted by frequency. The temporal phenotype graphs were embedded as vector representations, which were then used in support vector machine algorithms. Accuracies of area under the curve (AUC) = 0.73 and AUC = 0.72 were achieved for prediction of 1-year hospitalization after CHF and for early prediction of CHF, respectively, which were both higher than three alternative baseline methods. Different temporal phenotypes were identified for hospitalization and incident CHF, with differing disease and medication “hubs” for each phenotype.

### 5.3 Unipartite patient-patient similarity network

Another approach to patient phenotyping involves developing unipartite patient–patient networks, also known as a patient similarity network (PSN). These networks consist of only patient nodes that are extracted from the bipartite patient–disease graph based on their shared diseases. The shared edges of the PSN are created based on the same similarity algorithms described for the creation of the DCN. Novel graph-based features can be generated for each patient node, including community membership, once a community detection algorithm has been applied to the PSN. These communities can be considered to reflect clinical phenotypes since the modules will be based on patients with a common set of shared diseases. In addition to clustering using community detection algorithms, clustering can also be performed based on only the node’s (patient) properties, including demographic information and clinical characteristics. The resulting set of communities from the different medical domain data can then be used for downstream tasks, including risk prediction. Alternatively, separate PSNs can be used as input for heterogeneous graph neural networks (46).

The development of a PSN was used for the prediction of future diabetes, with 38 communities detected from a weighted unipartite projection with edge weights inversely proportional to the degree (number of connections) of each node (62). The network modularity of 0.57 with an average clustering coefficient of 0.808 reflected highly connected communities (35). Although cluster membership was not used in the prediction models, eigenvector centrality, closeness centrality, and the clustering coefficient were important predictors of diabetes, indicating that the similarity of patients based on shared diseases can assist with diabetes diagnosis. When predicting LOS in older patients with chronic disease, the K-NN algorithm was applied to a PSN created using the Jaccard similarity score to detect the K = 100 nearest neighbors. For each node (patient), aggregated LOS functions (mean, SD, min, and max) were then calculated based on their neighbors and used with baseline information and features from a DCN to predict LOS (59). The PSN features accounted for 33.1% of the feature importance for LOS prediction using a random forest algorithm that achieved an *R*^2^ = 0.347.

### 5.4 Bipartite similarity network

**Figure 5:**
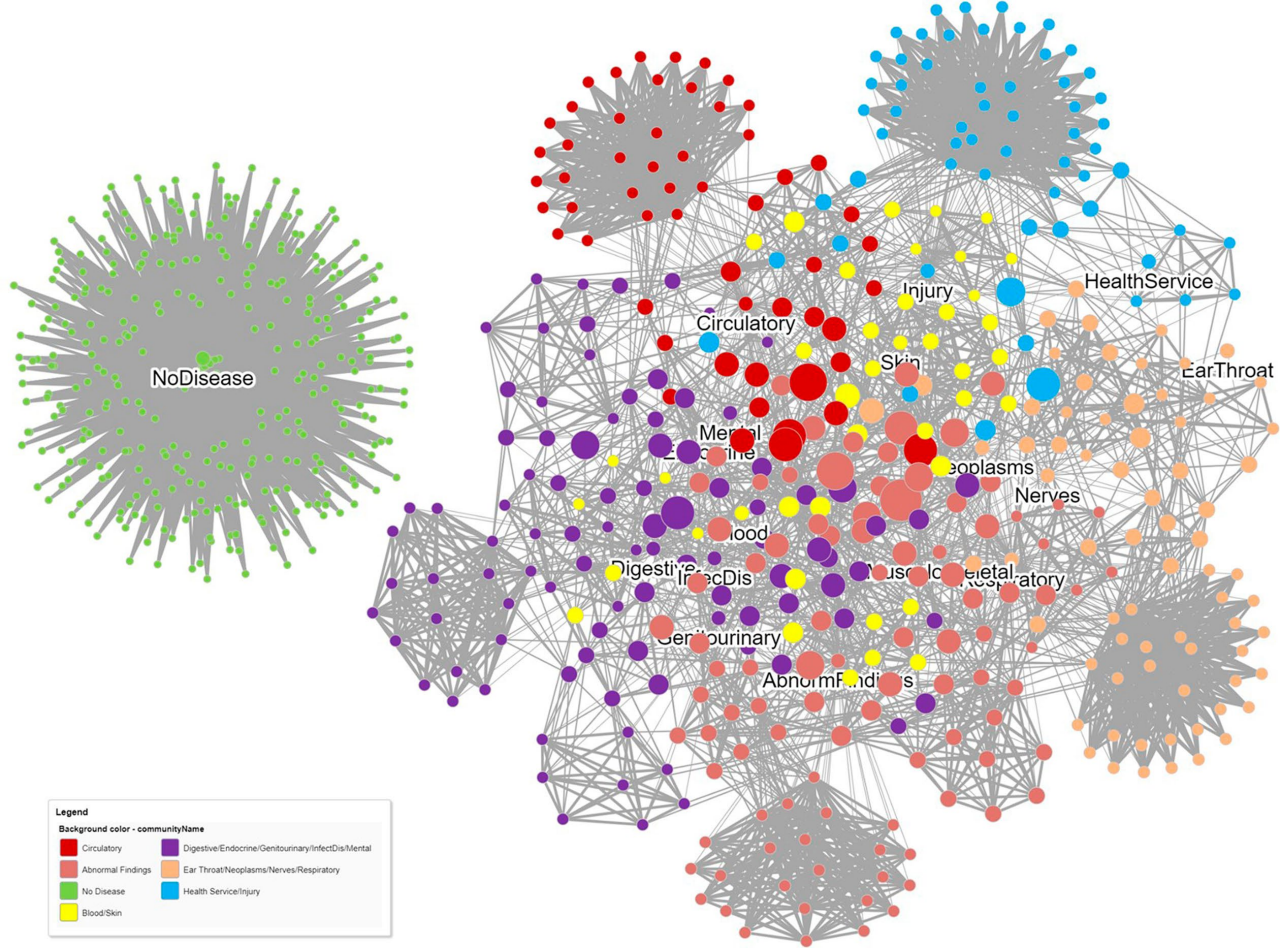
A dual relationship network obtained from projecting both patient–patient similarity relationships and patient–disease relationships. Patient similarity is reflected by the darkness of the patient–patient edges. Node sizes indicate the number of diseases each patient has. Communities were detected using the Louvain community detection algorithm.

A third network that projects the patient–patient similarity relationships and the patient–disease relationships can be created as a basis for phenotyping. Community clusters are formed for patients and diseases together, allowing meaningful naming of the patient clusters. In Figure 5, a patient similarity network and a patient–disease network are combined into a single graph, and a community detection algorithm is then applied to identify modules. The level of patient similarity is reflected by the darkness of the patient–patient edges; patients in the center of the network are generally less similar than those at the periphery of the network. Node sizes indicate the number of diseases each patient has. Some patients form clusters related to a single disease. Patients with blood or skin diseases (yellow dots) all have more than one disease chapter diagnosis and are centered toward the center of the network and spread around many different diseases.

## 6 Graph feature extraction algorithms

Beyond node similarity and community detection algorithms, many other graph algorithms exist that can be used for developing novel features from networks that reflect the importance and influence of nodes in a network. These graph features can then be used for downstream prediction with ML classification algorithms.

### 6.1 Graph centrality algorithms

Centrality algorithms determine the importance of nodes in a network in relation to their influence in maintaining the structure of the network and their relevance to information flow across the network. They include measures of centrality (degree, betweenness, closeness, and eigenvector) and page and article rank (see Box 3 for mathematical definitions of common centrality algorithms). More detailed definitions of centrality algorithms have been described elsewhere (33).

The structural basis of a system is often loosely defined as being either a hierarchical, random, or scale-free network (29) with the latter defined by the degree distribution having a power-law tail such that P(k) ~ k^–γ^, where γ is called the degree exponent. In the context of multimorbidity, a scale-free network suggests the existence of central disease “hubs” that provide stability to the network and likely play a key pathogenic role in disease progression (63). Formal testing for the existence of a scale-free network can be performed using Vuong’s test to compare log-normal, exponential, and Poisson distributions and to determine the likely existence of disease “hubs” that could be targeted for intervention (28), an idea supported by the observation that node centrality measures are often strong predictors of health outcomes. For example, degree centrality, eigenvector centrality, closeness centrality, and betweenness centrality from a unipartite patient network defined by their shared diseases were each significant predictors of incident diabetes (35). Similarly, highly connected diseases in a COPD comorbidity network were strongly associated with important patient-related outcomes, including mortality, pulmonary rehabilitation, quality of life, acute exacerbations, and hospitalization (30). The “hubs” identified in a network will likely vary according to the level of disease classification used (three-digit vs. four-digit ICD-9 codes) as well as the degree of adjustment used in selecting the edges to be retained (51). In an intensive care unit (ICU) patient network, the top 10 nodes by degree were very different in networks that did or did not adjust for other conditions when calculating edge strength odds ratios (64).

As a rule-of-thumb, researchers sometimes refer to the 20% of nodes in a network with the highest degree as the “hubs,” although this is an arbitrary definition since a scale-free property implies that such networks do not have any inherent threshold beyond which nodes are “hubs” (29). When examining the structure of COPD networks, the latter were found to be scale-free in comparison to non-COPD patients, highlighting the existence of centrally important diseases within the COPD network (30). Specifically, approximately one-third of the comorbidities possessed two-thirds of the edges.

BOX 3 **Common centrality algorithms.**

**Degree centrality**

Degree centrality, also known as the node of a degree, is the simplest measure of node centrality and is a count of the number of nodes linked to the node. It can be interpreted as the ability of a node to catch and to propagate information flow through the network. A normalized form of the degree centrality is computed as:

Normalized degree centrality (u) =$\frac{degree\;\left(u\right)}{N-1}$

where *N* is the size of the network (number of nodes).

**Eigenvector centrality**

Eigenvector centrality rests on the concept of a node being more important if it has important neighboring nodes since connections to these influential nodes will increase the influence of the given node. The influential effect is modeled by making the degree of each node proportional to the average centralities of its neighbors. For the adjacency matrix A, where $A_{uv}$= 1 if node u is connected to node v, the eigenvector centrality for node u is:

$$\mathrm{Ax = x, u = 1, 2}\;\lambda x_{u}=\overset{n}{\underset{v=1}{\sum }}A_{uv}x_{v},$$

where, λ is a unique positive eigenvalue.

**Closeness centrality**

According to closeness centrality, a node is crucial if it has small, shortest-path lengths to all other nodes. The centrality closeness of the node u, C*_c_* (u), is defined as:

$$C_{c}\mathrm{(u)}=\frac{1}{\sum_{v\in N}d\;\left(u,v\right)}$$

where, *N* is the set of nodes in the network and d (u, v) is the shortest-path length between u and v.

**Betweenness centrality**

Betweenness centrality considers a node as important when it lies on many shortest-paths between other nodes. The betweenness centrality of the node u, C*_b_* (u), is defined as:

$$C_{b}\mathrm{(u)}=\underset{s\neq v\neq t\;}{\sum }\frac{\sigma_{st}\left(u\right)}{\sigma_{st}}$$

where, $\sigma_{st}\left(u\right)$ is the number of shortest-paths between s and t that contain u, and $\sigma_{st}$ is the shortest-path between s and t.

### 6.2 Closeness and betweenness centrality algorithms

Closeness centrality indicates how closely a node is linked to all other nodes and therefore reflects the degree of likely contagion of a disease to other comorbid diseases. Betweenness centrality evaluates how many shortest paths a particular node has between pairs of other nodes. Nodes with a high betweenness centrality are often called bottlenecks (29). In the context of comorbidity, diseases with high betweenness are also strong candidates for targeted therapeutic interventions since they act like bridges connecting other diseases and are likely to increase the multimorbidity burden of patients.

### 6.3 Eigenvector centrality algorithms

Diseases with high eigenvector centrality are those conditions related to more influential diseases, which may help in indicating which disease pairs are causally related (34). When predicting hospital LOS from a multimorbidity network (MN) of older patients, an eigenvector centrality (EVC) score for patients obtained by summing the EVC of their disease nodes was an important factor in predicting LOS, improving the *R*^2^ by 18.7% beyond patient clinical and demographic data (59). In a DCN with 120 communities including nine major disease groups, EVC scores improved overall accuracy, sensitivity, and specificity, which were 69.52, 78.81, and 69.02%, respectively, for predicting high-cost patients (32). Degree centrality, eigenvector centrality, closeness centrality, betweenness centrality, and the clustering coefficient, from separate unipartite patient–patient and disease–disease networks, together considerably improved prediction accuracy for diabetes (AUC = 0.911) (35). Eigenvector centrality (measuring patient influence in the network), closeness centrality (measuring the closeness to other patients), and age had the highest Gini feature importance.

### 6.4 Aggregated network features

It is also informative to describe a network using graph parameters that capture the overall size, topology, and complexity of the network. These include the total node count, total edge count, modularity, number of communities, network diameter, graph density, average degree, path length, and clustering coefficient. The graph density in an undirected network is the total number of edges divided by the total number of possible edges, indicating the degree of possible transition between nodes. It is useful for comparing network structures and can be compared using a *z*-score test with bootstrapped or jack-knife standard errors created using resampling of the graph vertices (64). Network diameter is the average number of edges between two nodes, and average path length is a measure of node closeness obtained by measuring the shortest path between a node and all other nodes in the graph. Each of these parameters was described when creating a patient similarity network to predict the risk of type 2 diabetes (35). Average path length, average degree, and network diameter were determined when developing different multimorbidity networks across the lifespan (34). In COPD and CHF multimorbidity disease–disease networks, different graph densities were obtained for men and women (0.249 and 0.180, respectively) as well as different average degrees (25.9 and 17.5, respectively), demonstrating the high, although still differing, level of connectivity of diseases for patients in these populations (28). Similarly, the network density for COPD patients displayed unique disease–disease links and was much higher than that of non-COPD patients, with 79 nodes and 428 links versus 56 nodes and 149 links (30). Other measures used to describe bipartite networks (with nodes U and V) include linkage density D = L(|U| + |V|), connectance (the fraction of all possible links (L) that are realized, C = L/(|U| × |V|)), generality G = L/|U|, vulnerability V = L/|V|, and web asymmetry W = (|V|–|U|)/(|U| + |V|) (33).

## 7 Graph embedding (graph representation learning)

Traditional ML and deep learning techniques generally perform well when applied to medical data due to the regular tabular data structure, which provides high translational invariance to new data. However, graphs, including patient and disease networks, are typically irregular in shape and high-dimensional. To become suitable for analysis, a graph must therefore be transformed into fixed-dimensional vectors that can be used as new features for node and edge prediction. Graph representation learning aims to obtain low-dimensional vector representations of graph entities (e.g., nodes, edges, subgraphs, etc.) whilst preserving graph structure, semantics, and entity relationships, which requires specifying non-linear transformation functions (20). Thus, the embedding is optimized to ensure that nodes with similar network neighborhoods are also close in the vectorial space (and algebraic operations performed in this learned space reflect the network’s topology). In biological networks, this also reflects the local hypothesis that, for example, highly similar pairs of protein embeddings suggest similar phenotypic consequences. Similarly, the shared-components hypothesis requires that two nodes with significantly overlapping sets of neighbors should have similar embeddings, owing to shared message passing with, for example, highly similar disease embeddings implying shared disease-associated cellular components (20). Graph embedding models include graph kernels, matrix factorization-based models, shallow models, as well as deep neural network models, and non-Euclidean models that allow end-to-end prediction using the graph as input data (65) (Figure 1).

### 7.1 Graph kernels

Graph kernels were an early method used to learn graph embeddings by considering the similarity of surrounding nodes. Graph kernels aim to compare graphs or their substructures (e.g., nodes, subgraphs, and edges) by measuring their similarity, which is what lies at the core of the unsupervised learning of graphs. There are several strategies to measure the similarity of pairs of graphs, such as graphlet kernels, WL kernels, random walk, and shortest paths. The main idea of graphlet kernels is to count the number of different graphlets with the same size in a graph (65, 66).

### 7.2 Matrix factorization-based models

Although graph kernels work well on small graphs, they have limitations in learning node embeddings when working with large and complex graphs. Matrix factorization models are based on singular value decomposition to find eigenvectors in the latent space, thereby reducing the high-dimensional matrix of graphs (e.g., adjacency matrix, Laplacian matrix) into a low-dimensional space. The advantages of matrix factorization-based models include the small data requirements to learn embeddings in comparison to other methods, such as neural network-based models. They also provide good graph coverage for the proximity of all nodes in the graph. However, the computational complexity of matrix factorization is high for large graphs with millions of nodes due to the time it takes to decompose the matrix into a product of small-sized matrices. Importantly, models based on matrix factorization cannot handle incomplete graphs with unseen and missing values, and matrix factorization-based models can also not learn generalized vector embeddings, which are required for node and edge prediction of new data.

### 7.3 Shallow models (DeepWalk, Node2vec)

Shallow models involve compression of the N × N adjacency matrix of the N graph nodes into 2-D embedding vectors (an N × 2 matrix) using a neural network with a single hidden layer. Larger real-world networks with millions or even billions of nodes will typically have more than two dimensions (128–256 or higher) to represent larger real-world graphs. This approach provides a much lower dimensional feature space and an effective solution for graph-related downstream tasks. Various shallow models have been proposed to learn embeddings with different strategies to preserve graph structure. These typically implement a sampling technique to capture graph similarity, a Euclidean distance function to measure embedding similarity, and an optimization procedure such as a shallow neural network that minimizes the loss function between the graph and embedding similarity functions (20). DeepWalk and Node2Vec were two pioneer models to use shallow neural networks and allow preservation of the node neighborhoods based on random walk sampling, which could capture global information in graphs (65). The main idea of the random walk strategy is to gather information about the graph structure to generate paths that can be treated as sentences in documents. A graph node neighbor is randomly selected, a walk is made to that neighbor, and this continues until sufficient node sequences are obtained. The distances between node representations in the embedding space correspond to the frequency with which a particular node is visited in random walks originating from another node. The random pathways are converted into sequences, which are then clustered into similar nodes. Due to its purely random nature, DeepWalk had limitations in capturing graph structure, which were resolved using Node2vec, which used a biased random walk sampling process with two parameters (p and q) to adjust the random walks. This allowed the model to capture more information on the graph structure both locally and globally by introducing constraints when deciding on the subsequent nodes visited.

### 7.4 Non-Euclidean models

Most existing graph embedding models aim to learn embeddings in Euclidean space, which may not deliver good geometric representations and metrics. Recent studies have shown that non-Euclidean spaces are more suitable for representing complex graph structures. The non-Euclidean models can be categorized as hyperbolic, spherical, and Gaussian (65).

## 8 Graph machine learning

Shallow embedding methods are termed transductive algorithms since although they capture the semantics of domain data to offer a defined interpretation, they can only learn and return embedded values for their training data. Obtaining the embedding vector for unseen data is not possible. Shallow models such as DeepWalk and Node2Vec also mainly work well on homogeneous graphs and generally ignore information about the attributes/labels of nodes that could be informative for graph representation learning. Inductive node embedding algorithms include graph neural networks (GNNs) and non-GNNs. The latter include GraphSAGE, FastRP (using random projection and matrix operations), and HashGNN (hashing function architecture).

### 8.1 Graph neural networks

Graph neural networks (GNNs) are a deep learning family of models introduced in 2005 after it was hypothesized that since information can be represented naturally using graphs, it should be possible to process graph structure data directly rather than using the traditional approach of node embedding, in which information may be lost (67). However, since the aim of GNNs is to aggregate the information from graph structures, which consist of non-Euclidean data structures, GNNs still borrow ideas and methods from graph embedding and also from convolutional neural networks, in which the data are passed through a series of layers to learn new representations (68). Graph embeddings in GNNs are generated via (neural) message passing over a series of propagation layers; each layer passes neural messages based on messages passed in the previous layer. This is followed by the aggregation of messages among neighboring nodes and the updating of representations, in which a non-linear transformation is applied using the aggregated message and the embedding from the previous layer. A myriad of GNN architectures define different messages, aggregation, and update schemes to derive deep graph embeddings (20).

In contrast with methods for shallow network embedding, GNNs generate representations of the graph components that capture the graph network topology and the node features (69) enabling fully end-to-end prediction of node properties, edges, clustering, and similarity. GNNs also capture higher order and non-linear patterns through multi-hop propagation within several layers of neural message passing. Their weaknesses include high complexity, scaling difficulties, and low interpretability and explainability. The current research and application domains of GNNs have considerably increased in the last 12 years due to the growing interest in graph structure data mining (65) and they have more recently become more widely used in graphical analysis due to their excellent performance. In medicine, GNNs are seen as an emerging field for medical diagnosis, treatment, and disease prediction (22). Examples of their use to date include predicting drug–drug interactions (43), modeling polypharmacy side effects, and learning the temporal patterns of disease development in comorbidity networks (47). In the latter, the mapping of patient histories to edge weights to model temporal representations of disease trajectories enabled the simultaneous prediction of diseases and a better understanding and representation of disease pathology. Several forms of graph NNs now exist, including graph convolutional networks (GCN) that induce informative latent feature representations of nodes. The embedded vectors of each node are the transformed and weighted sum of the feature vectors of its neighbors. The deeper the network, the larger the neighborhoods, such that global information rather than purely local information is disseminated to each graph node to learn better node embeddings. Other graph NNs include graph attention networks (GAT), graph isomorphism (GIN), JK-Net (jumping knowledge network), and message passing neural networks (MPNN) (70) that are designed to integrate existing medical data with known medical ontologies.

#### 8.1.1 Example: predicting self-harm

A disease–disease comorbidity network of 938 diseases was combined with patient information and a novel Diagnosis to Vector (Dx2vec) embedding model to develop a deep neural network (DNN) for predicting self-harm (40). The comorbidity network was created using 2,323 self-harm cases and 46,460 controls for a 1:20 ratio. The embedding model simultaneously represented the diagnoses, the comorbidity patterns among diagnoses, and the temporal patterns of historical inpatient admissions for each patient as low-dimensional feature vectors. The DeepWalk embedding algorithm was first used to capture the closeness of diseases in the network, and max-pooling was then used to capture the most distinct features of the embedded diseases at each episode. These embedding vectors are then fed into a long short-term memory (LSTM) unit to learn the final Dx2vec embedding and capture both the temporal patterns of multiple inpatient admission episodes and the topology of the comorbidity network. The Dx2vec embedded vector was concatenated with the indicators of diagnoses, age, and gender of the patients, and this final vector was then fed into a deep neural network (DNN) to generate a risk prediction model for self-harm in the next 12 months. An accuracy of AUC = 0.89 compared with a baseline DNN that did not have access to the graph network of AUC = 0.85. The sensitivity of the Dx2vec and baseline models were 0.72 and 0.65, respectively.

### 8.2 Heterogeneous graph NNs

Although GNNs can be applied to disease comorbidity networks to learn their structural nature, such graphs are homogeneous, consisting of only disease nodes, and fail to capture the heterogeneous nature of medical data, which includes demographic information, laboratory results, medication prescriptions, medical imaging, and text from patient note codes. Heterogeneous medical domain graphs consist of different medical entities connected by multiple relationship types to enable the merging of data from different sources and the creation of a continuous flow of information.

#### 8.2.1 Example: disease prediction

The ability to predict separate ICD-9 disease diagnosis codes in ICU patients was examined using a heterogeneous graph similarity neural network (HSGNN) (46). A heterogenous graph consisting of multiple medical entities is first transformed into multiple similarity subgraphs using the different meta-paths (visit–disease–visit, medication–visit–patient, disease–visit–medication) contained within the initial overall heterogeneous graph. From the separate subgraphs, a new graph is learned using similarity matrices and meta-path importance from the subgraphs. In this way, the structural information relating to relationships between medical entities present in the original graph was maintained, and the initial separation into homogeneous graphs also prevented the over-smoothing of the data. Finally, the new overall graph is fed into the GNN. The HSGNN outperformed other baseline GNN models when applied to more than 46,000 patients in the MIMIC-III dataset, improving the AUC for ICD-9 classification disease diagnosis at both the patient level and at the visit level (46).

#### 8.2.2 Example: diabetes prediction

The clinical diagnosis of diabetes was modeled by building a multi-relational graph using patient demographics, laboratory features, medications, and the interactions between them, as well as two other graphs based on node characteristics and the higher order semantics of the nodes. These three graphs were then combined into a heterogeneous network (with multiple node and edge types), which was jointly optimized using GNNs in disease prediction (71). The model markedly improved the AUC for diabetes prediction from 76% using a standard GNN to 92%, demonstrating that division of the multi-relational graph into separate components could create a higher order semantic graph that incorporates complex interactions between medical entities and improves disease prediction.

#### 8.2.3 Example: a heterogeneous GNN for online disease diagnosis based on symptoms

A heterogeneous GraphNN named the Healthcare Graph Convolutional Network (HealGCN) harnessed the complex interactions between users, symptoms, and diseases in EHR data to develop a disease diagnosis service for online users, including primary care doctors and patients (72) that incorporated a graph-based symptom retrieval system (GraphRet) to provide a list of relevant alternative symptoms. The model showed around a 5% improvement in accuracy compared to baseline models including GraphSAGE and Med2Vec, which ignore the complex interaction types between nodes.

#### 8.2.4 Example: a heterogeneous graph for predicting adverse drug reactions

A heterogeneous GNN was developed to improve the prediction of post-marketing adverse drug reactions by learning node representations of a heterogeneous drug–disease graph from 12 years of healthcare claims data (73). The GNN aggregated the information of each drug/disease node, and the weighted sum of neighboring node features in previous GNN layers were used as node features for subsequent layers. The performance of the algorithm for predicting drug–ADR pairs was superior to that of a logistic regression model and neural network (AUC = 0.795 vs. 0.631 and 0.739, respectively). Combining several forms of the algorithm also predicted ADRs not present in the database.

## 9 Knowledge graphs

### 9.1 Knowledge graphs for precision medicine

A knowledge graph (KG) has been defined as a graph of data intended to accumulate and convey knowledge of the real world, whose nodes represent entities of interest and whose edges represent the different relations between these entities (74). The term knowledge graph was first coined by Google in 2012 when they developed them for use in their next-generation search engines, which recognized not only the objects in a search but also the relationship between them (75). In addition to being widely adopted for use in natural language processing tasks (76), KGs are used for varied purposes in the biomedical domain, including studying gene interactions, disease phenotypes, drug interactions, patient diagnoses, and patient–treatment predictions (20). In addition to combining information across different medical domains, including drugs, genes, proteins, and diseases, an important advantage of using KGs in the context of precision medicine is their inherent ability to constrain the vast solution space when dealing with multimodal health data for prediction (29, 77). KGs can also reveal insights into the pathology of diseases since disease comorbidity reflects the shared molecular mechanisms or environmental factors between diseases (27). For example, KGs were used to make novel gene–disease predictions for autism spectrum disorder (24). Within multimorbidity, KGs have the potential to accelerate a precision medicine approach to healthcare by efficiently combining knowledge from multiple datasets, including those relating genes, proteins, molecules, drug compounds, and diseases, to develop a better understanding of comorbidities or specific diseases. By further integrating patient clinical records into networks, graph representation learning of EHRs, and knowledge databases, we can generate predictions for disease and treatments tailored to individual patients that reduce the risk of ADRs (20, 73, 78).

#### 9.1.1 Example: treatment recommendation with reduced adverse drug reactions

To customize medication recommendations for patients with complex health conditions and reduce drug–drug interactions, the Graph Augmented Memory Network (GAMENet) integrated a drug–drug knowledge graph with longitudinal patient EHR data. It was trained end-to-end using a GCN to provide both more effective and safer personalized recommendations, including a reduction in drug–drug interactions from 7.5 to 3.9% (78). GAMENet also outperformed baseline models in predicting a patient’s current set of treatments (AUC = 0.69) among 1,058 test patients from the MIMIC-III dataset receiving an average of 14 medications.

#### 9.1.2 Example: adverse drug reaction prediction

The detection of ADRs was developed using 12 years of healthcare claims data to create a heterogeneous KG of prescription and disease codes in combination with a GNN. Proximity-based node embedding was obtained for the drugs and diseases using the Skip-gram model, which also captured temporal sequences. This was fed to a GNN that leveraged multilayer message passing to predict ADRs (73). Newly described drug–ADR pairs were predicted with high probability (0.972–0.985).

#### 9.1.3 Example: medication recommendation

Shallow embedding models were used for medication recommendation by developing a network of MIMIC-III patients, medicines, and medical knowledge (ICD-9 ontology and DrugBank). Recommendations were generated based on link predictions for a bipartite patient–medicine projection with the top-ranked medications selected for treatment (79). Compared to three baseline models, the KG achieved the highest prediction accuracy (0.611) and the lowest drug–disease interaction rate (0.17%).

#### 9.1.4 Example: patient diagnosis

An automated knowledge graph was created from EHR medical notes relating to diseases and symptoms to improve patient diagnosis (80). There were 156 diseases and 491 symptoms generated as medical concepts from the ED data of 273,174 patients. Compared to clinician expert opinion, the KG had a precision of 0.87 at a recall of 0.50 for detecting disease–symptom edges. The KG also surpassed the recall of the Google Health Knowledge Graph (GHKG), suggesting that the new graph detected relevant symptoms not suggested by the GHKG.

### 9.2 Open-source knowledge graphs

Many large-scale open-source disease-related knowledge graphs have now been generated using publicly available datasets, some of which are available to researchers as open access resources. These include PrimeKG (precision medicine knowledge graph) (81), Hetionet (heterogeneous network) (82), HINGRL (heterogeneous information network graph representation learning) (44) and SPOKE (scalable precision medicine open knowledge engine) (83). These have been applied for drug repurposing, detecting drug contraindications, discovering relationships between diseases and other related entities, including genes, proteins, and drug compounds, and for disease prediction.

*PrimeKG* is a knowledge graph designed to provide a holistic and multimodal view of diseases, using the networked relationships from different biological scales to support research into human disease and precision medicine (81). PrimeKG integrates 20 different publicly available resources describing more than 17,000 diseases and over 4 million relationships representing 10 major biological scales, including disease-associated protein perturbations, biological processes and pathways, anatomical and phenotypic scales, and the entire range of approved drugs with their therapeutic action. PrimeKG identified an abundance of indications, contradictions, and off-label drug–disease edges (81).

*Hetionet* is a heterogeneous network using data from 29 publicly available biomedical sources, with 11 node types (including compounds, genes, proteins, diseases, and symptoms) and 24 relationship types (including compound–disease, compound–gene, and gene–disease) (82). The complete KG consists of 47,031 nodes, 1,552 compounds, and 136 diseases. Hetionet was used to calculate the probability of a compound being a candidate treatment for diseases across 209,168 different compound–disease pairs. The degree-weighted path count (DWPC) was used to estimate the prevalence of compound–disease paths. Of 29,044 non-treatments (compounds not currently used to treat a disease), 1,206 were considered in a model for treatment, of which 709 were significant. An overall area under the receiver operating characteristic (AUROC) of 97.4% demonstrated high performance in detecting known treatments, and the same model performed well in validation datasets (85.5 and 70.0%). Examples for epilepsy and nicotine dependence verified the high ranking of existing treatments and clearly showed the properties that made other non-treatments likely candidates for drug repurposing. Whilst the original focus of Hetionet was for drug repurposing, the network also identifies the biological processes involved in specific diseases, the drug targets responsible for causing specific side effects, and anatomies with transcriptional relevance for a specific disease.

*HINGRL* considers both network topology and biological knowledge to identify new indications for drugs by integrating drug–disease, drug–protein, and protein–disease biological networks with the biological knowledge of drugs and diseases (44). Different representation strategies were applied to learn the features of the nodes in the heterogeneous information network from topological and biological perspectives. When used to predict unknown drug–disease associations based on these integrated drug and disease features, HINGRL outperformed three other state-of-the-art algorithms proposed for drug repositioning, with an AUC of 0.8835 and 0.9363 using separate benchmark datasets.

*SPOKE* is a heterogeneous biomedical knowledge graph developed as a basis for enabling a precision medicine approach to treatment, which connects patient EHRs with information from laboratories, procedures, and diagnoses to a knowledge network to provide real-world patient context (84). EHRs from 878,479 patients were used to develop 3,233 medical concepts, including 137 diseases, which were overlapped with the 47,000 nodes in the knowledge network using a random walk algorithm. The importance of each node was determined based on the time spent on any node during the walk, and this information is then stored in embedded vectors called propagated spoke entry vectors (PSEVs). The study demonstrated the ability of the PSEVs to recover deleted disease–disease, disease–gene, compound–compound, and compound–gene edges as well as infer new relationships between side effects and anatomy nodes. SPOKE now connects information from 41 biomedical databases and contains more than 21 node types and 55 edge types (83).

In an updated version of SPOKE, with 400 K knowledge nodes and 7,535 SEPs, SPOKE was again embedded into EHRs using the same modified version of the PageRank algorithm to uncover the hidden patterns of information existing between the concepts in the patient records and the knowledge nodes (85). The PSEVs improved prediction of multiple sclerosis (MS) for 5,752 patients 3 years before diagnosis (AUC = 0.83 vs. AUC = 0.60 using only EHRs) and provided insight into the biological drivers of MS. The same SPOKE KG was used for the early detection of Parkinson’s disease (86) with AUC accuracies of 0.77, 0.74, and 0.72 for 1, 3, and 5 years before diagnosis, respectively, and accuracies of 0.74, 0.70, and 0.66 in a validation cohort. These were all higher at each time point than when only EHRs were used (0.67, 0.63, and 0.56 at 1, 3, and 5 years, respectively).

### 9.3 Open-source graph databases

Many publicly available graph databases also exist for educational and benchmarking purposes, including the Network Repository Project (87) and the Open Graph Benchmark (OGB) (88) that provide a repository of graph datasets, allowing users to train their models in predicting nodes, edges, and subgraphs and to compare their performance against other algorithms. OGB contains a diverse set of challenging benchmark datasets that are large-scale (up to 100+ million nodes and 1+ billion edges) and include biological networks and knowledge graphs. The Harvard Dataverse is a general research dataset repository that contains graph databases, including the PrimeKG knowledge graph (81). The Integrated Complex Traits Networks (iCTNet) is an app and database that allows researchers to build heterogeneous networks by integrating a variety of biological interactions, thus offering a system-level view of human complex traits (77).

## 10 Conclusion

Experts involved in developing guidelines for treating patients with multimorbidity acknowledge that there exists an urgent need to transform the current approach to prescribing, which relies on guidelines developed for different populations without consideration of the potential for drug–disease interactions and polypharmacy that can result if applied to older patients with multimorbidity. The development of such guidelines for this population also requires using observational real-world data to adequately incorporate patient heterogeneity, in addition to borrowing information from existing biomedical knowledge databases. Real progress in this direction is now being achieved by researchers applying techniques from network analysis, graph ML, and open-source knowledge graphs, thereby creating the required basis for precision medicine approaches to treatment in this population. Our article provides an overview of some of these powerful techniques, along with examples of their application in the context of multimorbidity.

By developing disease comorbidity and patient similarity networks, an improved understanding of the structure of these networks is being achieved, as is the ability to transfer information from such graphs into formats that allow prediction of disease diagnosis and health outcomes. The use of network algorithms to identify disease hubs, significant network connections, and disease and patient phenotypes provides a way to identify the diseases that should be targeted for treatment to disrupt disease progression whilst also incorporating more holistic care that is based on the patient phenotype rather than on each individual disease. Fully end-to-end graph ML in both non-neural network and neural network-based forms allows inductive models that can predict outcomes and pathways on new data unseen by the original graph. These networks can be designed to utilize information from multiple clinical domains, including disease diagnoses, laboratory data, and patient reports. Knowledge graphs have been combined with medical concepts obtained from real-world health datasets to relate medical concepts to the knowledge of thousands of medical entities and have been shown to provide accurate treatment recommendations for patients whilst minimizing the risk of prescribing errors. In these various ways, graph algorithms, graph representation learning, graph neural networks, and knowledge graphs are providing the novel insights required to develop safe and holistic approaches to prescribing for older patients with multimorbidity.

Several important factors make network analysis especially suitable for addressing the issues involved in developing suitable precision medicine approaches for the management of multimorbidity. Differential treatment responses can be influenced by various aspects of the patient phenotype, which must be formally elicited using robust statistical methods, including adaptive signature design studies (89) to identify genetic signatures, and established community detection algorithms used in network analysis (53, 54) for overall patient phenotyping. Similarly, since disease case incidence and other health outcomes include random variability, analytical approaches are required that incorporate the stochastic nature of health events over time (90). Here, networks have proven useful for simultaneously representing the physiological interactions occurring within the human organism, identifying the primary mediators of information flow within that network, and detecting those regulated physiological variables that become widely disconnected over time in individuals with a poor prognosis. Finally, successful modeling for precision medicine typically requires an element of data reduction to capture patient phenotypes efficiently and accurately whilst using lower dimensional datasets. This may involve either feature selection methods, in which only the most relevant physiological variables are selected for use in prediction (91) or unsupervised clustering methods, in which a large number of informative features are reduced to a smaller set of cluster variables, including the modules identifiable using community detection algorithms.

It is also important to acknowledge the limitations of network analysis in relation to developing a precision medicine holistic approach to prescribing for the older multimorbid population and the need to consider how network analysis might integrate with other AI-based machine learning algorithms that are now being leveraged to assist with clinical decision support. For example, whilst network analysis can suggest new treatments and make predictions on health outcomes, the best treatment policy to apply for a particular patient must still be decided upon, and this requires determining from the potential treatment plan options the plan that provides the best health outcomes. Again, rapid progress is being made in AI machine learning fields such as reinforcement learning, which uses deep learning techniques to identify the best policy for long-term reward (92, 93). The use of reinforcement learning has already achieved success in other patient populations and medical settings, including treating sepsis within the intensive care unit (94), diabetes management (95) including optimization of glycemic control and blood pressure (36), optimizing hemodialysis for patients with anemia (37), and for prescribing in cancer (38).

It is therefore hoped that by combining different AI techniques including network analysis, to identify candidate treatments based primarily on patient phenotypes, with other state-of-the-art ML algorithms such as reinforcement learning or recommender systems, reliable personalized and holistic treatment plans can be determined for individuals with multimorbidity. This will allow for the provision of clinical decision support tools that can achieve optimal outcomes for a highly heterogeneous patient population with very differing levels of clinical complexity.

## Author contributions

RW: Conceptualization, Data curation, Formal analysis, Methodology, Visualization, Writing – original draft, Writing – review & editing. BK: Conceptualization, Writing – review & editing, Methodology. AM: Conceptualization, Writing – review & editing, Data curation, Resources.
