# Supplementary figures and images for: Applying precision medicine principles to the management of multimorbidity: the utility of comorbidity networks, graph machine learning, and knowledge graphs
Source: Front Med (Lausanne). 2024 Jan 24;10:1302844. doi: 10.3389/fmed.2023.1302844 (PMC10885565; doi:10.3389/fmed.2023.1302844)

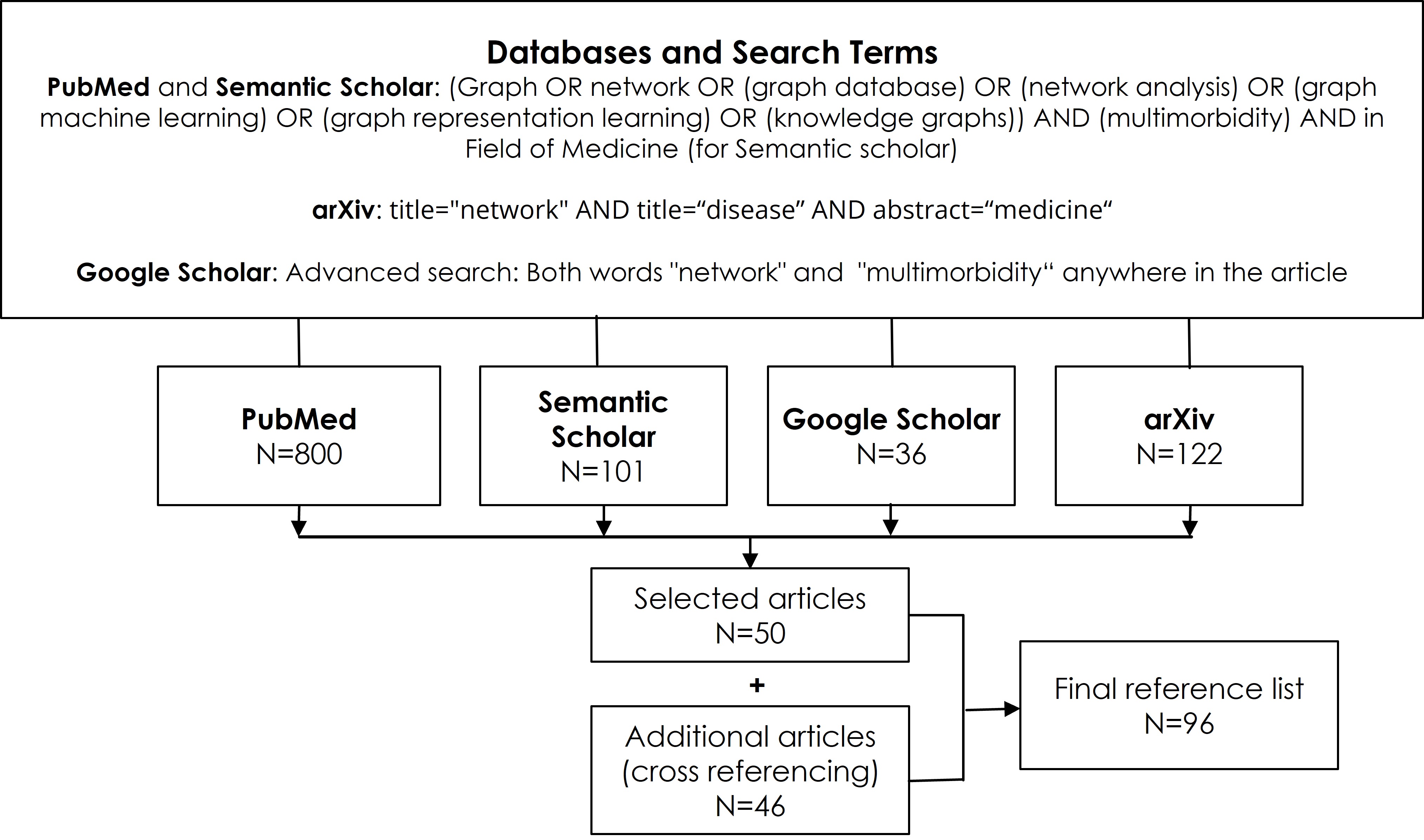

Supplement: Supplementary file 1 [file Image_1.jpg]
